# A sequential multi‐strain inoculation approach for designing functional Sicilian table olives

**DOI:** 10.1002/jsfa.70180

**Published:** 2025-09-11

**Authors:** Irene M Zingale, Amanda Vaccalluzzo, Giacomo Antonio Calandra Checco, Vita Maria Marino, Margherita Caccamo, Cinzia L Randazzo, Nunziatina Russo, Dilara Nur Dikmetas, Tuba Esatbeyoglu, Esra Capanoglu, Cinzia Caggia

**Affiliations:** ^1^ Department of Agriculture, Food and Environment University of Catania Catania Italy; ^2^ Consorzio per la Ricerca nel settore della Filiera Lattiero Casearia e dell'agroalimentare – CoRFiLaC Ragusa Italy; ^3^ ProBioEtna srl, Spin off University of Catania Catania Italy; ^4^ Department of Food Engineering, Faculty of Chemical and Metallurgical Engineering Istanbul Technical University Istanbul Turkey; ^5^ Department of Molecular Food Chemistry and Food Development, Institute of Food and One Health Gottfried Wilhelm Leibniz University Hannover Hannover Germany

**Keywords:** table olive fermentation, starter, *Lacticaseibacillus rhamnosus*, bile salt hydrolase, volatile organic compounds (VOCs)

## Abstract

**BACKGROUND:**

The exploitation of microbial strains with advanced technological traits is a pivotal step in enhancing the production of table olives and meeting consumers' demands.

**RESULTS:**

Here, a multi‐step approach involving the sequential addition of a *Lactiplantibacillus plantarum* (namely C11C8) starter strain and a *Lacticaseibacillus rhamnosus* (namely VB1) probiotic strain with confirmed bile salt hydrolase activity was applied for the production of Nocellara Etnea and Tonda Iblea cultivars, fermented at 7% NaCl. Results revealed that in olives inoculated with the starter, staphylococci and Enterobacteriaceae were found below detectable limits. Furthermore, fermentation significantly increased total phenolic content (TPC) and radical scavenging activity (RSA) in Tonda Iblea brine samples, with the highest values for TPC (310.13 μg gallic acid equivalent mL^−1^) and RSA (222.27 μmol Trolox equivalent mL^−1^) after 150 and 120 days, respectively. Furthermore, the presence of the probiotic strain, confirmed by quantitative PCR, resulted in a slight reduction of TPC, although a value of 219.63 μmol Trolox equivalent mL^−1^ in Nocellara Etnea brine samples was detected after 150 days. The volatile organic compound profiles revealed that in samples inoculated with the two strains, benzaldehyde and *α*‐farnesene, associated with fruity and floral notes, were detected. In contrast, the control samples exhibited higher levels of guaiacol and 4‐ethylphenol, known for imparting off‐flavors. Sensory analysis confirmed that consumers preferred olives enriched with the probiotic, for their aromatic complexity and reduced off‐flavors.

**CONCLUSION:**

The proposed approach results in a promising strategy for designing lower‐sodium functional table olives. © 2025 The Author(s). *Journal of the Science of Food and Agriculture* published by John Wiley & Sons Ltd on behalf of Society of Chemical Industry.

## INTRODUCTION

In recent years, the trend for healthier eating habits has placed fermented foods in the spotlight as rich sources of bioactive compounds.^1^ Among them, table olives, for their integral role in the Mediterranean diet, appear very interesting to function as carriers of health‐promoting bacteria. Modern biotechnological approaches have redefined these iconic fruits by integrating fermentation processes with functional food innovation, transforming olives into probiotic‐rich, plant‐based alternatives to traditional dairy products.^2^, ^3^ This paradigm shift aligns with increasing consumer demand for sustainable, cholesterol‐free and lactose‐free options. These attributes are supported by the high nutritional value of table olives, which are rich in fiber, vitamins, minerals, phenolic compounds, polyunsaturated fatty acids and antioxidants.^4^ Table olives are deeply rooted in the Mediterranean diet, with processing methods varying across countries like Spain, Greece, Italy and Portugal.^5^ World production for the provisional crop year 2023–2024 is estimated at 2 828 500 t, down 12% on the previous year,^6^ although between 2011 and 2021, EU countries produced approximately 833 090 t of table olives annually, with global production reaching 3.1 million tonnes and consumption at 2.95 million tonnes during the 2022–2023 season.^7^, ^8^

In Sicily, a distinctive processing method involves immersing olives directly in brine, allowing natural fermentation to occur through indigenous microbiota without the need for debittering pretreatments or starter cultures.^9^ This spontaneous fermentation, dominated by lactic acid bacteria (LAB) and yeasts, initiates a series of biochemical transformations and refines the sensory characteristics of the final products.^10^ To mitigate bitterness and inhibit the growth of pathogenic or undesirable microorganisms, table olives are traditionally treated with high‐sodium solutions.^11^ However, the World Health Organization (WHO) recommends a daily limit of sodium intake of 5 g to prevent health risks. This recommendation limits the consumption of high‐sodium olives and strongly emphasizes the need for safe production systems for table olives with reduced sodium content, while respecting traditional production.^12^, ^13^

The selection of LAB with advanced technological abilities has been a pivotal step in enhancing the production of table olives and meeting consumers' demands. Recent studies have demonstrated that starter cultures, particularly those represented by *Lactiplantibacillus plantarum* and *Lactiplantibacillus pentosus* species, can be useful for optimizing the fermentation processes.^14^ The single‐ and multifunctional starter cultures in table olive fermentation have become attractive for the food industry as they contribute to reducing the process time and spoilage risk.^10^ Controlled fermentation, using either autochthonous or allochthonous starters, ensures the safety and high quality of fermented fruits and vegetables.^15^ In naturally green table olive production, these microorganisms synergize with native microbiota to guarantee microbiological safety, accelerate bitterness removal and enrich the nutritional profile of the final product. These advancements are primarily related to the enzymatic degradation of oleuropein, a phenolic compound responsible for the bitterness in olives. *Lpb. plantarum* strains exhibit proficiency in bio‐converting oleuropein into bioavailable and lower‐molecular‐weight phenolic compounds, through *β*‐glucosidase and esterase enzymes.^16^, ^17^ Specifically, *β*‐glucosidase catalyzes the hydrolysis of oleuropein into oleuropein aglycone and/or decarboxymethylated oleuropein aglycone (HyEDA), while esterase cleaves oleuropein aglycone into elenolic acid and hydroxytyrosol. Subsequently, enzymatic activity converts HyEDA into ethylenediamine, mitigating bitterness and enhancing the functional properties of olives.^18^, ^19^, ^20^

Nowadays, consumers are increasingly attuned to the health benefits of food beyond basic nutrition, driving the rising trend toward fermented products.^1^ Among them, naturally fermented olives are known to host diverse health‐promoting strains in their native microbiota, highlighting their potential as probiotic carriers.^21^, ^22^ The addition of a strain able to show resilience under gastrointestinal conditions, to modulate gut microbiota, produce short‐chain fatty acids and inhibit pathogens can further enhance the functional properties of table olives.

In recent years, within the field of probiotics, there has been growing interest in selecting strains with bile salt hydrolase (BSH) activity, an enzyme naturally present in numerous LAB and closely associated with benefits for intestinal health and regulation of lipid metabolism.^23^ BSH activity allows the deconjugation of bile salts, reducing their reabsorption in the intestine and promoting their excretion. This mechanism promotes the hepatic synthesis of new bile acids from cholesterol, contributing to a reduction in serum cholesterol levels.^24^ BSH activity has been considered as a selection criterion for probiotic bacteria of many strains, particularly in LAB, including *Lacticaseibacillus rhamnosus*, *Lacticaseibacillus casei*, *Lactiplantibacillus plantarum*, *Limosilactobacillus fermentum*, *Limosilactobacillus reuteri* and *Lactobacillus acidophilus*.^25^, ^26^, ^27^

The use of BSH‐active strains, as an added culture, in table olives could be an innovative strategy to enhance their functional profile, in the context of cardiovascular health. Because of their unique microstructure, olives provide a favorable environment for the survival and delivery of probiotics along the gastrointestinal tract, making them an effective plant‐based alternative to traditional dairy‐derived probiotics.^2^, ^28^, ^29^

Finally, by addressing concerns regarding sodium content, probiotic‐enriched table olives can evolve from plant‐based fermented foods to functional foods, offering a sustainable solution that honors Mediterranean traditions and meets modern nutritional goals.

## MATERIALS AND METHODS

### Table olive production

**Figure 1:**
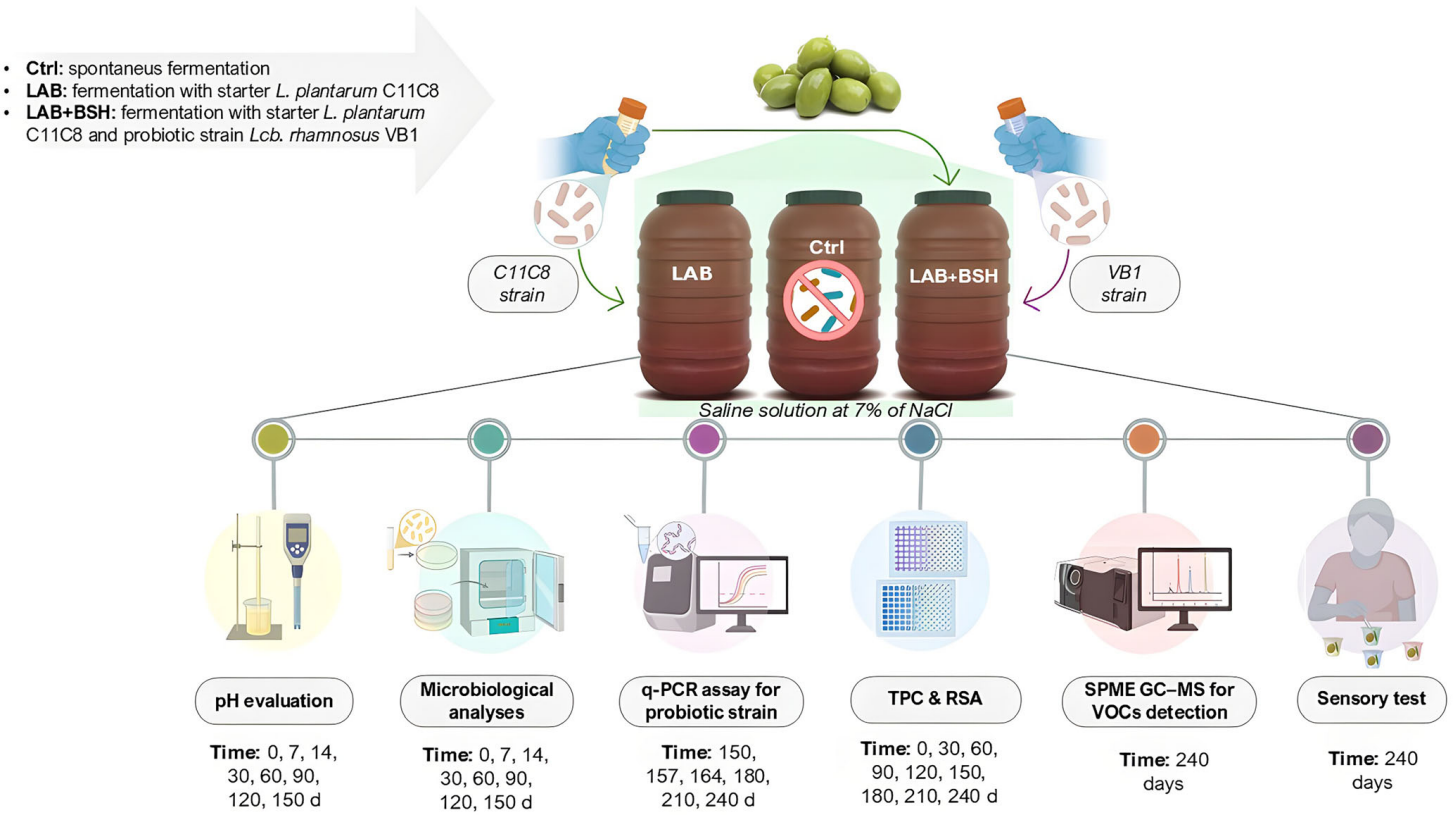
Experimental design of table olive fermentation trials with different inoculation strategies: Ctrl: spontaneous fermentation; LAB: fermentation with starter *Lpb. plantarum* C11C8; LAB+BSH: fermentation with starter *Lpb. plantarum* C11C8 and probiotic *Lcb. rhamnosus* VB1 strain. TPC: total phenolic content; RSA: radical scavenging activity.

Drupes belonging to Nocellara Etnea (Ne) and Tonda Iblea (Ti) cultivars were processed at the local company Azienda Agricola Morina Domenico (Paternò, Italy), according to industrial procedures. In detail, after harvesting (September–October 2023), the drupes, subjected to strict quality control, were thoroughly washed with water, graded and transferred to polyethylene containers (20 L volume) and directly immersed in a brine solution (7% NaCl w/v), with a ratio of olive to brine of 1:1. The olives were added with the strain *Lpb. plantarum* C11C8, belonging to the Di3A microbial culture collection, previously isolated from the brine of naturally fermented table olives (Fig. 1), selected for its ability to grow in brine, reducing the bitter compounds.^4^, ^17^, ^20^, ^25^ The strain was stored at −80 °C in de Man, Rogosa and Sharpe (MRS) medium (pH 6.5) containing 25% (v/v) glycerol, and grown in MRS medium, supplemented with 1.5% (w/v) agar, when required, and incubated at 37 °C for 24 h. Samples were inoculated with *Lpb. plantarum* C11C8, at a final cell density of 7 log CFU mL^−1^, and Ne LAB and Ti LAB samples were obtained, in triplicate. For both cultivars, controls were obtained without the addition of any starter (namely Ne Ctrl and Ti Ctrl). All fermentations were carried out at room temperature (18 ± 2 °C) and monitored for 150 days. During the fermentation time, marine salt was periodically added to maintain a constant sodium chloride concentration. After 150 days from the addition of *Lpb. plantarum* C11C8, olive samples of both cultivars were added with an *Lcb. rhamnosus* strain, namely VB1, from human origin, belonging to the microbial culture collection of ProBioEtna srl, Spin Off of the University of Catania, Italy.^30^ The strain, characterized for safety traits, according to the European Food Safety Authority (EFSA) (2021), previously characterized from a genotypic and phenotypic point of view, including antimicrobial resistance profile and adhesion ability, as reported by Agolino and co‐workers^26^ was selected for its high ability to deconjugate bile salts *in vitro*. The strain was supplied in lyophilized form by SACCO System srl and directly resuspended at a final cell density of 9 log CFU mL^−1^ in Ne and Ti brines previously fermented with *Lpb. plantarum* C11C8. Both brine and drupe samples were periodically collected and subjected to microbiological and chemical analyses.

The experimental design, as shown in Fig. 1, outlined the activities undertaken to monitor the fermentation process at both microbiological and chemical levels. This included evaluating pH, performing microbial count plate assays and conducting quantitative polymerase chain reaction (qPCR) detection of *Lcb. rhamnosus* VB1 at T150, T157, T164, T180, T210 and T240 (respectively at the time of inoculum and after 7, 14, 30, 60 and 90 days). Additionally, the total phenolic content (TPC) and radical scavenging activity (RSA) were assessed together with the detection of volatile organic compounds (VOCs) and sensory analysis.

### pH evaluation during fermentation

The pH values of the brine samples were measured after 0, 7, 14, 30, 60, 90, 120 and 150 days of fermentation, using a pH meter (Mettler DL25, Mettler‐Toledo, Milano, Italy).

### Microbiological analyses

Microbiological analyses were performed as previously reported^20^ on both brine and drupe samples before the addition of starter (T0) and after 7, 14, 30, 60, 90, 120 and 150 days of fermentation. In detail, 25 g of olives was taken, drained, pitted and transferred into a sterile stomacher bag containing 225 mL of Ringer's solution (1:10 w/v) and homogenized for 2–5 min in a stomacher. The brine and the homogenized olive samples were serially diluted and subjected to plate counting. In particular, plate count agar, incubated at 32 ± 2 °C for 48 h, was used for total mesophilic bacteria; MRS agar, supplemented with cycloheximide (5 mL L^−1^), anaerobically incubated at 32 °C for 24–48 h, for LAB count; Sabouraud dextrose agar, supplemented with chloramphenicol (0.05 g L^−1^), incubated at 25 °C for 4 days, for yeast counts; violet red bile glucose agar, incubated at 30 °C for 18–24 h, for Enterobacteriaceae counts; mannitol salt agar, incubated at 32 °C for 48 h, for enumeration of coagulase‐positive and ‐negative *Staphylococcus* spp.; Mac Conkey, incubated at 32 °C for 24–48 h, for enumeration of *Escherichia coli*; sulfite polymyxin sulfadiazine agar, anaerobically incubated at 37 °C for 24–48 h, for the detection of sulfite‐reducing clostridia, such as *Clostridium perfringens*; and *Bacillus cereus* agar (Mossel) incubated at 30 °C for 24 ± 2 h, for the detection of *Bacillus cereus* spp. Additionally, the presence of *Listeria monocytogenes* and *Salmonella* spp. was detected in Palcam agar incubated at 37 °C for 24–48 h, and in Hektoen agar incubated at 37 °C for 24 ± 2 h, respectively, following conventional procedures.^31^, ^32^ All media were purchased from Biolife (Milan, Italy). All analyses were performed in triplicate, and results were expressed as log_10_ CFU mL^−1^ for brine samples and log_10_ CFU g^−1^ for olive samples, and the standard deviation was given.

### Monitoring of probiotic strain survival by q‐PCR assay

To assess the persistence of *Lcb. rhamnosus* VB1, olive samples were taken and subjected to total DNA extraction at T150, T157, T164, T180, T210 and T240 using a Dneasy Mericon Food Kit (Qiagen, Milan, Italy) following procedures previously reported,^20^, ^33^ with slight modifications. In detail, 12 g of olive samples was diluted in 30 mL of sterile Ringer's solution and homogenized in a stomacher for 5 min at room temperature. The suspension was centrifuged at 10 000 × *g* for 10 min at 4 °C, and the pellet was washed twice with 30 mL of phosphate buffer solution, pH 7.4. Subsequently, the pellet was suspended in 400 mL of sterile Ringer's solution and transferred to a tube containing 0.3 g of zircon microspheres, to which 150 mL of phenol solution was added, and homogenized using a Precellys Evolution homogenizer (Bertin Technologies, Genova, Italy) at 10 000 rpm for 5 min. The resulting suspension was extracted following the kit's instruction manual. The concentration of extracted DNA was determined using a Qubit 4.0 fluorometer (Invitrogen, Carlsbad, CA, USA). The DNA was stored at −20 °C until further analyses.

To quantify the presence of *Lcb. rhamnosus* VB1, the extracted DNA was subjected to qPCR using strain‐specific primers, appropriately designed based on *in silico* studies of the sequences encoding the BSH gene.^34^ In detail, qPCR reactions were performed using a WizzPure SYBER Green Kit, using a Rotor Gene Q instrument (Qiagen, Milan, Italy). The qPCR reaction mixture was run in a final volume of 20 μL, containing 10 μL of SYBR green, 10 μmol L^−1^ of primers bsh_rha_qF2 (GGAATACGGGTGGCATACAA) and bsh_rha_qR2 (CAGGCCAAACATGCCATAAC), 5 μL of gDNA and 3.8 μL of ultrapure water. The amplification cycle included an initial denaturation step at 95 °C for 5 min, followed by 40 cycles at 95 °C for 30 s, 60 °C for 60 s and 60 °C for 30 s. To ensure the specificity of the reaction, a melting curve analysis was performed between 60 and 95 °C. To validate the reaction, a standard curve was generated using gDNA isolated from *Lcb. rhamnosus* VB1 strain, considering a concentration range from 9 to 3 log CFU mL^−1^. All reactions were performed in triplicate.

### Brine treatment for TPC and RSA determination

The TPC and the RSA were determined in brine samples at T0, T30, T60, T90, T120, T150, T180, T210 and T240. The extraction was performed following a method previously described^35^, ^36^ with some modifications. Specifically, 5 mL of 80% (v/v) ethanol was added, and samples were maintained in an ultrasonic bath for 15 min at room temperature. Then, samples were centrifuged at 4 °C and 4500 × *g* for 10 min, and supernatants were collected. Finally, the two supernatants were combined to obtain a final volume of 10 mL, then directly filtered (0.45 μm) and analyzed.

### TPC determination in olive brines

The determination of TPC was performed following the Folin–Ciocalteu method. In detail, 20 μL of each sample was injected in triplicate into 96‐well plates to which 100 μL of Folin reagent (0.2 N) and 100 μL of sodium carbonate solution (Na_2_CO_3_ 7.5%) were added. In addition, 100 μL of catechin hydrate solution (1 mg mL^−1^) diluted 1:10 was used as a positive control, and 100 μL of 99% ethanol was used as a negative control. The blue complex was visualized at 765 nm using a UV–visible spectrophotometer (Tecan Infinite® 200 PRO, Männedorf, Switzerland) after 60 min at room temperature. Gallic acid (2.5–100 μg mL^−1^) was used for constructing the standard curve, and the mean of three readings was used to determine the TPC expressed as μg of gallic acid equivalent (GAE) per mg of extract.

### RSA determination in olive brines

The 2,2‐diphenyl‐1‐picrylhydrazyl (DPPH) radical scavenging assay was performed to evaluate the RSA.^37^ In detail, a stock solution (1000 μmol L^−1^) was prepared by dissolving 9.86 mg of DPPH radical into 25 mL of 99% ethanol, and from the stock, a radical DPPH solution (300 μmol L^−1^) was diluted into ethanol. A total volume of 100 μL of each sample was injected in triplicate into 96‐well plates to which 100 μL of radical DPPH solution (300 μmol L^−1^) was added. The measurements were carried out at a wavelength of 515 nm using a UV–visible spectrophotometer (Tecan Infinite® 200 PRO, Switzerland) after 30 min at room temperature. Trolox (water‐soluble vitamin E derivative: 6‐hydrox‐*tert*‐2,5,7,8‐tetramethylchroman‐2‐carboxylic acid) at 5–25 μmol L^−1^ was used for calibration, and the antioxidant activity of each sample was expressed as Trolox equivalent (μmoL TE (100 g)^−1^). Ascorbic acid (1 mg mL^−1^) was used as a positive control, and 99% ethanol as a blank.

### VOC detection by SPME GC–MS in olive samples

Olive samples were collected at the end of fermentation (T240) and subjected to VOC detection using a gas chromatograph equipped with a mass spectrometer (GC–MS). After rinsing with distilled water to remove brine residues, olive samples were dried, stoned and homogenized at 5000 rpm for 18 s (Grindomix GM200, Retsch GmbH, Haan, Germany). Then 4.00 ± 0.02 g of pulp was weighed into 20 mL glass vials with PTFE screw caps and placed in a thermostatic block at 45 °C for 60 min with stirring. A DVB/CAR/PDMS‐coated solid‐phase microextraction (SPME) fiber (50/30 μm, Supelco, USA) was used for headspace adsorption of volatile compounds. The fiber was preconditioned at 250 °C for 1 h in a GC–MS system and reconditioned at 250 °C for 5 min before each analysis. The SPME fiber was exposed to the headspace of the vial for 30 min to adsorb the volatile compounds and then thermally desorbed at 250 °C for 10 min in the injector port of a GC (Agilent 7890A, Santa Clara, CA, USA) coupled to a MS (Agilent 5975C, Santa Clara, CA, USA). Separation was performed on an HP‐5 capillary column (30 m × 0.25 mm × 0.25 μm film thickness; Agilent Technologies, Santa Clara, CA, USA) with injections in splitless mode at 250 °C. The chromatographic conditions were chosen in accordance with Sánchez and co‐workers,^38^ with specific modifications to improve the separation of volatile compounds. The oven temperature program was as follows: initially at 35 °C for 3 min, then at a rate of 6 °C min^−1^ to 120 °C, followed by a rate of 30 °C min^−1^ to 240 °C, held for 3 min. Helium was used as carrier gas at a flow rate of 1.2 mL min^−1^ and a pressure of 14.9 psi. The mass detector was operated in a scan mode at 70 eV with a scan rate of 5.15 scans per second. Peak identification was performed by comparing the mass spectra obtained with those of the Wiley 175 library (Wiley & Sons, Inc., Weinheim, Germany).

### Sensory evaluation

Sensory evaluation was performed as previously reported^39^ and following the Social and Societal Ethics Committee principles. Olive samples were taken after 240 days and rinsed in water to remove excess salt. A total of 33 panelists (20 women and 13 men, aged between 20 and 57 years) participated in the sensory evaluation of the four experimental samples (Ne LAB, Ne LAB+BSH, Ti LAB and Ti LAB+BSH). Each panelist was trained at the Department of Agricultural, Food and Environment (Di3A) of Catania following the International Olive Council guidelines, with specific familiarization on sensory descriptors (bitterness, saltiness, acidity, texture) and defect identification. Before the sensory evaluation, panelists attended a dedicated seminar covering the sensory attributes, evaluation procedures and objectives of the study. The samples, labelled using a random three‐digit code, were served at room temperature using plastic plates. The panelists were offered unsalted crackers and mineral water between tasting each sample. The attributes that defined the sensory profiles of table olives included: visual characteristics (green color intensity), odor (intensity of green olive aroma and eventually off‐flavors), rheological characteristics (intensity of crispness), taste, intensity of bitterness and overall acceptability. The intensity of each sensory attribute was evaluated using a numerical scale from 1 (poor) to 5 (excellent). Overall acceptability was evaluated using a 10‐point scale from 1 (unsatisfactory) to 10 (excellent).

### Statistical analyses

One‐way analysis of variance (ANOVA) followed by a Tukey *post hoc* multiple‐comparisons test was applied to the pH values, TPC, RSA and microbiological and VOC data from three replicates. Assumptions of normality and homoscedasticity were verified before ANOVA application by the Shapiro–Wilk test and Levene's test, respectively. Differences were considered statistically significant at *P* < 0.05. Data analysis was performed using JMP software (SAS Institute, version 12.0.1). For the sensory analysis, a one‐way ANOVA test was applied with cultivar (Ti *versus* Ne) and strain (LAB *versus* LAB+BSH) nested within cultivar as fixed effects and panelist as a random effect. Means were compared using Student's *t*‐test. Statistical significance was attributed to *P* < 0.05. Analyses were performed using JMP statistical software (SAS Institute, NC, USA) v.12.0.1.

## RESULTS

### Physicochemical and microbiological traits

The brine concentration was maintained at 7% NaCl. Table 1 presents the pH values determined in the control and experimental samples. The results showed an initial difference between Ne and Ti samples (both control and inoculated), with the latter showing a lower starting pH value. From the T7, a progressive lowering of pH was observed in all experimental samples, with a higher decrease in all Ti samples. After 30 days, the pH of all samples stabilized, with a tendency for the Ti samples to reach lower values. During the whole fermentation process, significant differences emerged between Ne LAB and Ti LAB samples, with final pH values of 4.28 and 3.68, respectively, after 150 days. Such final values are consistent with the recommended limits to ensure the microbiological safety of the final product.

**Table 1:** Values of pH expressed as means and standard deviations detected in olive brines at different days of fermentation

Sample	pH							
	Days of fermentation							
	0	7	14	30	60	90	120	150
Ne Ctrl	6.65 ± 0.01^dB^	5.18 ± 0.01^dB^	4.85 ± 0.01^cB^	4.63 ± 0.20^cB^	4.52 ± 0.02^bB^	4.48 ± 0.01^bB^	4.39 ± 0.01^aB^	4.32 ± 0.02^aB^
Ne LAB	6.66 ± 0.02^eB^	5.24 ± 0.02^dB^	4.94 ± 0.02^cB^	4.64 ± 0.30^cB^	4.45 ± 0.02^bB^	4.41 ± 0.01^bB^	4.25 ± 0.01^aB^	4.15 ± 0.03^aB^
Ti Ctrl	6.52 ± 0.01^cA^	4.46 ± 0.02^bA^	4.14 ± 0.04^bA^	3.84 ± 0.02^aA^	3.81 ± 0.01^aA^	3.81 ± 0.02^aA^	5.81 ± 0.01^aA^	3.82 ± 0.02^aA^
Ti LAB	6.40 ± 0.01^dA^	4.36 ± 0.02^cA^	4.07 ± 0.03^cbA^	3.82 ± 0.02^bA^	3.74 ± 0.02^bA^	3.67 ± 0.02^abA^	4.62 ± 0.01^aA^	3.63 ± 0.01^aA^

Ne Ctrl, Nocellara Etnea spontaneously fermented; Ne LAB, Nocellara Etnea with *Lpb. plantarum* C11C8; Ti Ctrl, Tonda Iblea spontaneously fermented; Ti LAB, Tonda Iblea with *Lpb. plantarum* C11C8. ^a–d^ Different lowercase letters within the same row indicate statistically significant differences among treatments (*P* < 0.05); values labeled with ‘a’ represent the lowest values, while subsequent letters indicate increasing values. ^A–B^ Different uppercase letters within the same column indicate statistically significant differences among samples for the same parameter (*P* < 0.05); values labeled with ‘A’ represent the lowest values, while subsequent letters indicate increasing values.

### Microbial dynamics in drupe and brine samples

The dynamics of the main microbial groups (expressed as log CFU g^−1^ or log CFU mL^−1^ of three replicates ± standard deviation) counted in drupe and in brine samples during the fermentation are reported in Table 2. The LAB population detection in drupe samples showed an increased cell density until T14 in both cultivars, in inoculated and control samples. In detail, an initial increase of about 2.4 and 3.5 log units was detected after 7 days in Ne Ctrl and Ti Ctrl samples, respectively, whereas experimental samples showed a significant increase of about 4 log units for Ne LAB and of almost 8 log units for Ti LAB samples. From T30, a reduction in LAB concentration of about 1.0–1.7 log units was observed in all samples, remaining quite constant from day 90 up to the end of fermentation (T150).

**Table 2:** Main microbial groups (expressed as log CFU g^−1^ or log CFU mL^−1^ of three technical replicates ± standard deviation) in drupe and in brine samples during fermentation

Days of fermentation								
Drupe sample	0	7	14	30	60	90	120	150
**Lactobacilli**								
Ne Ctrl	4.47 ± 0.02^aA^	6.87 ± 0.04^dA^	7.92 ± 0.02^eB^	7.75 ± 0.06^eA^	6.04 ± 0.04^cA^	6.31 ± 0.03^cB^	5.13 ± 0.04^bA^	5.22 ± 0.03^bA^
Ne LAB	4.45 ± 0.05^aA^	8.04 ± 0.24^dB^	8.25 ± 0.06^eB^	7.82 ± 0.04^dA^	6.61 ± 0.03^cA^	6.12 ± 0.02^cA^	5.59 ± 0.08^bB^	5.52 ± 0.07^bA^
Ti Ctrl	3.43 ± 0.02^aB^	6.92 ± 0.03^dA^	7.09 ± 0.04^dA^	7.82 ± 0.04^eA^	7.27 ± 0.05^eB^	6.25 ± 0.06^cA^	5.73 ± 0.02^bC^	5.70 ± 0.02^bB^
Ti LAB	3.24 ± 0.06^aB^	7.95 ± 0.02^dB^	8.13 ± 0.04^eB^	8.08 ± 0.03^eB^	7.80 ± 0.02^dB^	6.41 ± 0.02^cB^	5.98 ± 0.02^bC^	5.82 ± 0.04^bB^
**Yeasts**								
Ne Ctrl	2.36 ± 0.06^cB^	5.65 ± 0.05^cB^	6.12 ± 0.08^dB^	6.09 ± 0.03^dB^	4.21 ± 0.07^aB^	4.11 ± 0.05^aB^	4.80 ± 0.04^bA^	5.11 ± 0.06^bB^
Ne LAB	2.30 ± 0.03^bA^	4.33 ± 0.03^bA^	5.19 ± 0.07^cA^	5.24 ± 0.08^cA^	3.67 ± 0.03^aA^	3.88 ± 0.11^aA^	5.71 ± 0.04^dB^	4.71 ± 0.03^bA^
Ti Ctrl	3.51 ± 0.03^bB^	5.11 ± 0.08^bB^	5.84 ± 0.05^dB^	5.84 ± 0.04^dB^	4.32 ± 0.03^aB^	4.16 ± 0.04^aB^	5.55 ± 0.05^cdB^	4.96 ± 0.04^bB^
Ti LAB	3.54 ± 0.03^bA^	4.13 ± 0.09^bA^	4.90 ± 0.03^cA^	4.74 ± 0.34^cA^	3.61 ± 0.03^aA^	3.95 ± 0.04^abA^	5.59 ± 0.06^dB^	4.52 ± 0.04^bcA^
**Mesophilic bacteria**								
Ne Ctrl	3.48 ± 0.04^aA^	7.13 ± 0.07^cdB^	8.18 ± 0.03^eA^	8.57 ± 0.07^eB^	7.50 ± 0.03^dB^	6.80 ± 0.04^cbB^	6.38 ± 0.02^bB^	6.52 ± 0.03^bB^
Ne LAB	3.57 ± 0.02^aA^	5.51 ± 0.04^bA^	8.32 ± 0.03^eA^	8.26 ± 0.04^eB^	7.43 ± 0.05^dB^	6.37 ± 0.04^cA^	6.04 ± 0.45^cB^	5.48 ± 0.03^bA^
Ti Ctrl	4.50 ± 0.02^aB^	6.90 ± 0.04^dB^	6.93 ± 0.04^dB^	8.50 ± 0.03^eB^	6.55 ± 0.04^cA^	6.73 ± 0.04^cdB^	5.77 ± 0.03^bA^	5.73 ± 0.02^bB^
Ti LAB	4.62 ± 0.04^aB^	5.63 ± 0.04^cA^	8.10 ± 0.02^eA^	7.97 ± 0.07^eA^	6.17 ± 0.06^dA^	6.31 ± 0.05^dA^	5.64 ± 0.04^bA^	5.21 ± 0.04^bA^
**Staphylococci**								
Ne Ctrl	2.58 ± 0.02^bA^	4.22 ± 0.03^cC^	4.48 ± 0.04^cC^	4.81 ± 0.03^dC^	3.16 ± 0.04^bC^	2.28 ± 0.02^aA^	2.31 ± 0.02^aA^	nd
Ne LAB	2.61 ± 0.02^bB^	1.09 ± 0.04^aA^	1.02 ± 0.01^aA^	nd	nd	nd	nd	nd
Ti Ctrl	2.57 ± 0.03^bA^	3.26 ± 0.04^cB^	3.51 ± 0.04^cB^	3.56 ± 0.04^cA^	2.02 ± 0.02^aA^	2.61 ± 0.03^bA^	2.48 ± 0.02^bA^	nd
Ti LAB	2.67 ± 0.03^bB^	1.17 ± 0.02^aA^	1.02 ± 0.02^aA^	nd	nd	nd	nd	nd

Days of fermentation								
Brine sample	0	7	14	30	60	90	120	150
**Lactobacilli**								
Ne Ctrl	nd	7.26 ± 0.04^dA^	7.78 ± 0.03^eB^	7.35 ± 0.04^dA^	6.10 ± 0.02^cA^	5.12 ± 0.04^aA^	6.03 ± 0.02^cA^	5.84 ± 0.06^bA^
Ne LAB	6.52 ± 0.03^bcA^	7.37 ± 0.04^dA^	8.08 ± 0.03^eB^	7.87 ± 0.04^eB^	6.23 ± 0.07^bA^	5.51 ± 0.02^aA^	6.38 ± 0.05^bA^	6.88 ± 0.02^cB^
Ti Ctrl	nd	7.50 ± 0.07^cdB^	7.31 ± 0.05^cA^	7.17 ± 0.05^cA^	6.25 ± 0.03^aA^	6.43 ± 0.04^aB^	6.68 ± 0.04^bB^	6.52 ± 0.03^abA^
Ti LAB	6.22 ± 0.44^aA^	7.68 ± 0.04^dB^	7.96 ± 0.05^dB^	7.87 ± 0.04^deB^	6.60 ± 0.03^bB^	6.59 ± 0.01^abB^	6.93 ± 0.05^cB^	7.12 ± 0.07^cB^
**Yeasts**								
Ne Ctrl	nd	4.25 ± 0.05^aB^	6.21 ± 0.07^bB^	6.22 ± 0.04^bA^	7.74 ± 0.05^dC^	6.21 ± 0.07^bB^	7.35 ± 0.05^cB^	7.17 ± 0.05^cB^
Ne LAB	nd	3.23 ± 0.10^aA^	6.10 ± 0.10^cA^	7.17 ± 0.04^eB^	5.63 ± 0.04^bA^	5.20 ± 0.05^bA^	6.89 ± 0.02^dA^	6.71 ± 0.02^dA^
Ti Ctrl	nd	5.67 ± 0.08^aC^	6.32 ± 0.04^bB^	6.47 ± 0.04^bA^	6.40 ± 0.03^bB^	6.72 ± 0.05^cB^	7.58 ± 0.03^dB^	7.67 ± 0.04^dB^
Ti LAB	nd	4.62 ± 0.04^aB^	5.71 ± 0.02^bA^	7.06 ± 0.07^dB^	5.95 ± 0.07^bA^	5.87 ± 0.06^bA^	6.57 ± 0.03^cA^	6.39 ± 0.07^cA^
**Mesophilic bacteria**								
Ne Ctrl	4.81 ± 0.03^aA^	7.28 ± 0.07^dA^	7.92 ± 0.06^eB^	7.45 ± 0.03^dA^	6.28 ± 0.02^cB^	6.61 ± 0.03^cC^	6.07 ± 0.05^bcA^	5.91 ± 0.03^bB^
Ne LAB	7.16 ± 0.04^cC^	7.16 ± 0.04^cA^	7.51 ± 0.03^dA^	7.68 ± 0.04^dB^	6.31 ± 0.03^bB^	5.58 ± 0.03^aA^	5.98 ± 0.06^bA^	5.17 ± 0.03^aA^
Ti Ctrl	6.12 ± 0.05^aB^	7.91 ± 0.06^dB^	7.67 ± 0.03^cdB^	7.45 ± 0.02^cA^	6.05 ± 0.05^aA^	6.46 ± 0.04^bC^	6.48 ± 0.03^bB^	6.55 ± 0.02^bC^
Ti LAB	7.07 ± 0.05^cC^	7.51 ± 0.03^dB^	7.41 ± 0.03^cdA^	7.71 ± 0.03^dB^	6.03 ± 0.06^aA^	6.19 ± 0.02^aB^	6.39 ± 0.02^bB^	6.23 ± 0.03^abC^
**Staphylococci**								
Ne Ctrl	2.31 ± 0.02^aA^	6.40 ± 0.02^fB^	5.48 ± 0.02^eC^	4.68 ± 0.03^dB^	3.28 ± 0.01^bB^	3.32 ± 0.03^bB^	3.83 ± 0.03^cB^	nd
Ne LAB	3.23 ± 0.05^bB^	6.10 ± 0.07^dB^	4.38 ± 0.03^cB^	4.62 ± 0.04^cB^	2.48 ± 0.03^aA^	nd	nd	nd
Ti Ctrl	3.69 ± 0.03^bC^	5.85 ± 0.03^dA^	5.11 ± 0.03^cC^	5.17 ± 0.02^cC^	3.28 ± 0.02^bB^	2.20 ± 0.02^aA^	2.26 ± 0.04^aA^	nd
Ti LAB	3.91 ± 0.02^bC^	5.81 ± 0.03^cA^	3.29 ± 0.03^aA^	4.17 ± 0.04^bA^	2.72 ± 0.04^aA^	nd	nd	nd
**Enterobacteriaceae**								
Ne Ctrl	3.87 ± 0.03^bB^	4.08 ± 0.03^cbA^	5.79 ± 0.03^cB^	4.82 ± 0.04^dC^	3.59 ± 0.02^bA^	2.49 ± 0.03^aA^	nd	nd
Ne LAB	3.17 ± 0.04^aA^	4.73 ± 0.03^cB^	5.41 ± 0.03^dB^	3.56 ± 0.02^aB^	nd	nd	nd	nd
Ti Ctrl	3.65 ± 0.06^cB^	4.14 ± 0.03^dA^	4.59 ± 0.03^dA^	3.68 ± 0.03^cB^	3.07 ± 0.05^bA^	2.08 ± 0.04^aA^	nd	nd
Ti LAB	3.96 ± 0.05^bC^	4.10 ± 0.06^bA^	4.34 ± 0.03^cbA^	3.17 ± 0.03^aA^	nd	nd	nd	nd

Ne Ctrl, Nocellara Etnea spontaneously fermented; Ne LAB, Nocellara Etnea with *Lpb*. *plantarum* C11C8; Ti Ctrl, Tonda Iblea spontaneously fermented; Ti LAB, Tonda Iblea with *Lpb*. *plantarum* C11C8. ^a−f^ Different lowercase letters within the same row indicate statistically significant differences among treatments (*P* < 0.05); values labeled with ‘a’ represent the lowest values, while subsequent letters indicate increasing values. ^A–C^ Different uppercase letters within the same column indicate statistically significant differences among samples for the same parameter (*P* < 0.05); values labeled with ‘A’ represent the lowest values, while subsequent letters indicate increasing values. nd, not detected.

Regarding yeast populations, a significant increase of about 4 log units was observed in control olive samples starting from T30. In the inoculated samples (Ne LAB and Ti LAB), an increase of about 4.0 and 1.0 log CFU g^−1^, respectively, was noted. From T60 to T90, a non‐significant reduction in yeast was recorded in the controls, while inoculated samples showed a decrease of approximately 1 log unit, highlighting the acidification effect of the matrix by the addition of *Lpb. plantarum* C11C8 starter. The mesophilic bacteria count showed a significant increase in all samples from T7 to T30, followed by a reduction at the end of fermentation (T150), which was higher in inoculated samples than in controls. Throughout the fermentation process, mesophilic bacteria reached average values of around 5.0–6.0 log CFU g^−1^in all samples.

Focusing on microbiological results in brine samples, no LAB was detected in controls, while inoculated samples (Ne LAB and Ti LAB) exhibited an average concentration of 6.5 log units. Starting from T7, a significant increase was observed in all samples, with the inoculated samples (especially Ti LAB) reaching a concentration of 7.9 log CFU mL^−1^ within T30. After 30 days, a slight decrease in LAB was observed in all samples, which remained quite constant until the end of the process (T150), with values ranging between 5.9 and 7.0 log units. As for yeasts, they were absent at the beginning of fermentation in all brine samples. From T7, a rise in cell concentration was observed, particularly in inoculated samples, up to T30. After T60, a decrease in yeast populations was observed in inoculated samples, with average values between 6.3 and 6.7 log CFU mL^−1^, while brines of control samples showed an increase, reaching values of 7.1–7.3 log units. Similarly, the mesophilic bacteria count in brine samples was higher in inoculated samples than in controls. From T7 to T30, inoculated samples showed an increase in mesophilic bacteria, followed by a decrease in the subsequent days, with average values of 6.0 and 5.7 log units in control and inoculated samples, respectively.

Microbiological analyses for the detection of spoilage and pathogenic microorganisms, performed on both olives and brines, generally revealed that the introduction of the microbial starter positively influenced microbiological safety by reducing the presence of potentially pathogenic and altering microorganisms.

Regarding staphylococci, the initial concentration in olive samples was found to be about 2.6 log CFU g^−1^ in all samples. In the control samples (Ne Ctrl and Ti Ctrl), a progressive increase of 2.2 and 1.0 log CFU g^−1^, respectively, was observed, indicating a higher proliferation in samples without LAB addition. As a matter of fact, in inoculated samples (Ne LAB and Ti LAB), a significant decrease was observed as early as T7, reaching levels below the detection limit (<1 CFU g^−1^) by T150. A similar trend was observed in brine samples, where a decrease in staphylococci was detected starting from T90 in inoculated samples, while in control samples, comparable levels were achieved at T150.

For Enterobacteriaceae, an initial concentration of 6.1 log CFU mL^−1^ in brine samples was detected. In inoculated samples, they were found undetectable from T60 onward, whereas in the control samples, an initial increase was observed, peaking at T14 (5.79 log CFU mL^−1^, Ne Ctrl), followed by a gradual decrease down to 4.7–5.3 log CFU mL^−1^ by the end of fermentation. In contrast, in olive samples, no Enterobacteriaceae were detected at any sampling points (Table 2). *Escherichia coli*, sulfite‐reducing bacteria, *Bacillus cereus*, *Listeria monocytogenes* and *Salmonella* spp. were not detected in any tested samples (both in olive and brine samples) at any sampling times.

### Detection of *Lcb. rhamnosus* VB1 strain by qPCR

To confirm the *Lcb. rhamnosus* VB1 persistence, qPCR was performed. In detail, the threshold limit was calculated based on the distribution of standard curve values, with values between 9 and 3 log CFU mL^−1^. The validity of the reaction was confirmed from a slope value of −3.312, coefficient of determination (*R* ^2^) of 99.9% and reaction efficiency value (*E*) of 100.4%. In Table 3, the values of the quantitative reactions, expressed as log CFU mL^−1^, are reported.

**Table 3:** Detection of *Lcb. rhamnosus* VB1 in olive samples expressed as log CFU mL^−1^ (mean ± standard deviation)

Days of sampling	Ne LAB+BSH	Ti LAB+BSH
150	3.26 ± 0.03	2.88 ± 0.15
157	7.79 ± 0.05	6.83 ± 0.73
164	7.84 ± 0.08	6.47 ± 0.83
180	7.83 ± 0.25	7.25 ± 0.09
210	7.58 ± 0.28	7.48 ± 0.24
240	6.53 ± 0.41	7.36 ± 0.16

At T150, all samples showed a value of 3 log CFU mL^−1^. However, 7 days after inoculation (T157), a sudden increase in values, higher than 7 log CFU mL^−1^, was observed in the Ne LAB+BSH sample, which maintained similar values until T210, slightly decreasing at T240, with an average of around 6.5 log CFU mL^−1^. Similar values were also detected in the Ti LAB+BSH sample, with a value of 7 log CFU mL^−1^ up to T240.

### TPC and RSA in brine samples

Results of TPC in brine samples during fermentation (from T0 to T150) and after the inoculum of the BSH probiotic strain (from T180 to T240) are reported in Table 4. The results indicate a progressive increase in TPC during fermentation. In the case of the Ne cultivar, the spontaneously fermented samples (Ne Ctrl) achieved higher TPC values, compared to samples with starter (Ne LAB) during the first 150 days (Table 4). However, starting from T150, the Ne LAB samples exhibited a slight reduction in TPC, compared to the control. In contrast, the Ne LAB+BSH samples displayed a significantly higher final TPC than both Ctrl and LAB samples. Focusing on the Ti cultivar, Ti LAB samples maintained more constant TPC levels, compared to Ti Ctrl, whereas Ti LAB+BSH showed significantly higher TPC values (Table 4).

**Table 4:** Total phenolic content (μg GAE mL^−1^) detected in olive brines during fermentation

Sample	Days of fermentation								
	0	30	60	90	120	150	180	210	240
Ne Ctrl	9.34 ± 0.97^aB^	215.6 ± 1.08^bA^	240.11 ± 1.71^cA^	249.64 ± 1.02^cdA^	277.02 ± 0.75^gA^	308.21 ± 1.14^gA^	267.42 ± 1.92^bC^	266.93 ± 1.56b^cD^	254.31 ± 1.5^aA^
Ne LAB	13.9 ± 0.53^aB^	260.75 ± 1.28^dcC^	262.99 ± 1.46^dA^	251.25 ± 1.03^cdA^	244.16 ± 1.64^cA^	262.74 ± 0.66^dB^	161.01 ± 1.55^aA^	233.65 ± 1.86^bA^	276.82 ± 1.7^cB^
Ne LAB+BSH	‐	‐	‐	‐	‐	‐	192.82 ± 1.56^aA^	279.94 ± 0.86^bD^	282.18 ± 1.2^bC^
Ti Ctrl	7.29 ± 1.56^aA^	275.07 ± 0.92^dC^	281.07 ± 1.76^dB^	297.4 ± 0.63^eB^	292.16 ± 0.55^eB^	289.15 ± 1.31^deB^	244.53 ± 1.37^aC^	250.67 ± 1.21^aB^	260.15 ± 1.6^bA^
Ti LAB	5.86 ± 1.29^aA^	242.2 ± 1.38^cB^	279.87 ± 1.38^deB^	303.29 ± 1.07^fB^	294.41 ± 1.88^eB^	310.13 ± 1.82^fA^	218.38 ± 1.18^aB^	258.86 ± 1.69^bB^	274.36 ± 1.1^cB^
Ti LAB+BSH	‐	‐	‐	‐	‐	‐	229.04 ± 1.22^aB^	273.75 ± 0.69^bD^	280.25 ± 1.7^bC^

Ne Ctrl, Nocellara Etnea samples spontaneously fermented; Ne LAB, Nocellara Etnea with *Lpb*. *plantarum* C11C8; Ne LAB+BSH, Nocellara Etnea with *Lpb*. *plantarum* C11C8 + probiotic *Lcb. rhamnosus* VB1; Ti LAB, Tonda Iblea samples spontaneously fermented; Ti LAB Tonda Iblea with *Lpb. plantarum* C11C8; Ti LAB+BSH: Tonda Iblea with the addition of *Lpb. plantarum* C11C8 + probiotic *Lcb. rhamnosus* VB1. ^a−c^ Different lowercase letters within the same row indicate statistically significant differences among treatments (*P* < 0.05); values labeled with ‘a’ represent the lowest values, while subsequent letters indicate increasing values. ^A–D^ Different uppercase letters within the same column indicate statistically significant differences among samples for the same parameter (*P* < 0.05); values labeled with ‘A’ represent the lowest values, while subsequent letters indicate increasing values.

Regarding the RSA (as measured by the DPPH assay), a similar trend was observed, with an overall increase during fermentation. Results obtained during fermentation (from T0 to T150) and after the addition of the BSH probiotic strain (from T180 to T240) are reported in Table 5. In the Ne cultivar, the Ne Ctrl maintained relatively stable antioxidant levels after 30 days, whereas the Ne LAB+BSH samples showed an increase over time, reaching the highest antioxidant capacity value. In the Ti cultivar, Ti LAB exhibited a more pronounced antioxidant peak compared to Ti LAB+BSH. Furthermore, the addition of *Lcb. rhamnosus* BV1 (Ti LAB+BSH) preserved and significantly enhanced the antioxidant levels (Table 5).

**Table 5:** Radical scavenging activity (μmol TE mL^−1^) in olive brines expressed as mean and standard deviation at different times of fermentation

Sample	Days of fermentation								
	0	30	60	90	120	150	180	210	240
Ne Ctrl	55.14 ± 1.08^aB^	220.25 ± 0.98^fB^	220.72 ± 0.59^fB^	216.41 ± 1.86^dA^	215.09 ± 1.69^cA^	215.95 ± 1.20^cdA^	218.54 ± 1.69^aC^	216.71 ± 0.99^aB^	217.80 ± 1.21^aC^	
Ne LAB	45.23 ± 1.09^aB^	215.25 ± 0.66^dB^	214.63 ± 0.44^cA^	216.80 ± 1.50^deA^	218.14 ± 0.87^eA^	219.63 ± 1.91^efB^	134.97 ± 1.12^aA^	224.23 ± 1.73^bC^	221.21 ± 1.10^bC^	
Ne LAB+BSH	—	—	—	—	—	—	215.31 ± 1.22^bC^	206.93 ± 1.21^aA^	210.62 ± 1.32^bB^	
Ti Ctrl	29.18 ± 1.18^aA^	209.73 ± 0.76^bA^	215.64 ± 1.47^cdA^	217.52 ± 1.52^dB^	222.27 ± 1.03^eB^	215.19 ± 1.20^cA^	201.58 ± 1.39^aB^	219.98 ± 1.73^bB^	216.62 ± 1.61^bC^	
Ti LAB	32.49 ± 1.59^aA^	215.98 ± 1.75^cdB^	217.62 ± 0.66^dAB^	220.16 ± 1.56^eB^	218.57 ± 1.64^deA^	219.63 ± 1.91^eB^	195.32 ± 1.00^aB^	210.37 ± 1.08^abA^	200.33 ± 1.22^abA^	
Ti LAB+BSH	—	—	—	—	—	—	205.38 ± 0.98^bB^	191.21 ± 0.82^aA^	202.61 ± 1.23^abA^	

Ne Ctrl, Nocellara Etnea samples spontaneously fermented; Ne LAB, Nocellara Etnea with *Lpb*. *plantarum* C11C8; Ne LAB+BSH, Nocellara Etnea with *Lpb*. *plantarum* C11C8 + probiotic *Lcb. rhamnosus* VB1; Ti LAB, Tonda Iblea samples spontaneously fermented; Ti LAB Tonda Iblea with *Lpb. plantarum* C11C8; Ti LAB+BSH: Tonda Iblea with the addition of *Lpb. plantarum* C11C8 + probiotic *Lcb. rhamnosus* VB1. ^a−b^ Different lowercase letters within the same row indicate statistically significant differences among treatments (*P* < 0.05); values labeled with ‘a’ represent the lowest values, while subsequent letters indicate increasing values. ^A–C^ Different uppercase letters within the same column indicate statistically significant differences among samples for the same parameter (*P* < 0.05); values labeled with ‘A’ represent the lowest values, while subsequent letters indicate increasing values.

### Volatile organic compounds

The VOC detection in fermented olive samples led to the identification of 40 compounds, classified into different chemical groups, as: organic acids (3), esters (7), alcohols (5), aldehydes (9), phenols (6), lactones (1), heterocyclic compounds (2), aromatic hydrocarbons (2), terpenes (3) and other molecules (1), as reported in Table 6. The results revealed significant qualitative and quantitative differences between inoculated samples, both with LAB and with LAB+BSH, and control samples. Overall, in control samples, 27 compounds were detected, whereas in samples inoculated with starter and in samples inoculated with starter plus probiotic, 22 and 34 compounds were revealed, respectively, clearly highlighting the effect of probiotic strain on VOC profiles. Focusing on the two cultivars, a noticeable difference was found, with Ctrl samples showing 21 and 17 compounds, respectively, for Ne and Ti. Furthermore, these differences are accentuated in the samples inoculated with LAB and BSH, for which an inversion of the aromatic structure has been recorded, with the Ne exhibiting 15 compounds and the Ti samples as many as 24. In detail, the VOC profile of samples treated with starter (Ne LAB and Ti LAB) showed an increase of propionic acid and octanoic acid ethyl ester. In Ne LAB, the 2‐methoxy‐4‐methylphenol level was found to be 7.27% while in the Ne Ctrl the value was 12.17%. Ne samples inoculated with LAB+BSH showed enrichment of aromatic aldehydes, such as benzaldehyde (5.78%) and phenylethyl alcohol (6.56%), responsible for fruity and floral notes (Table 6). Notably, in Ne LAB + BSH, the combination of esters and aldehydes contributed to a more refined and complex aroma profile, with a concurrent reduction in off‐flavor compounds. Moreover, a higher concentration of *α*‐farnesene (3.13%), a terpene associated with pleasant floral attributes, was detected. In contrast, spontaneous fermentations resulted in less predictable VOC profiles, often characterized by higher levels of phenols, such as guaiacol (32.91%) in Ti samples and 4‐ethylphenol (3.58% and 7.1% in Ne Ctrl and Ti Ctrl, respectively) which impart undesirable smoky and harsh notes (Table 6), with the latter found to a lesser extent in Ti LAB and even lower in Ti LAB +BSH. Against the high levels of propanoic acid found in control and LAB‐inoculated samples, reduced levels were recorded in samples inoculated with starter plus probiotic, contributing to a more balanced aroma profile. In Ti samples treated with the probiotic strain (Ti LAB+BSH), an overall increase in 9‐octadecenoic acid and butanoic acid, 4‐hydroxy was observed, with guaiacol levels decreased to 21.6%, compared to 32.91% found in the control sample (Ti Ctrl). Focusing on alcohol content, a general decrease in samples inoculated with LAB or with LAB +BSH was observed compared to spontaneously fermented samples, with the notable exception of phenylethyl alcohol, which was found at higher levels in Ti samples, in Ctrl, in LAB and in LAB +BSH with values of 25.93%, 27.44% and 22.78%, respectively, contributing positively to the aroma profile (Table 6). In addition, Ti Ctrl showed a high 4‐ethylphenol content (7.51%) that was found at lower concentrations in Ti LAB and Ti LAB+BSH, at 3.33% and 2.99%, respectively.

**Table 6:** Volatile organic compounds, expressed as mean ± standard deviation, in Nocellara Etnea and Tonda Iblea olives after 240 days

Compound (area as %)	Ne			Ti		
	Ctrl	LAB	LAB + BSH	Ctrl	LAB	LAB + BSH
Acids						
Propanoic acid	57.60 ± 2.52^b^	63.68 ± 3.11^c^	54.47 ± 0.64^a^	4.39 ± 0.39^a^	5.63 ± 0.17^b^	4.84 ± 0.63^a^
Butanoic acid, 4‐hydroxy‐	n.d.	n.d.	n.d.	n.d.	n.d.	0.52 ± 0.18^a^
9‐Octadecenoic acid, (*E*)‐	n.d.	n.d.	n.d.	n.d.	n.d.	9.21 ± 0.56^a^
Esters						
Propanoic acid, ethyl ester	n.d.	13.61 ± 1.33^b^	6.21 ± 0.34^a^	n.d.	n.d.	n.d.
Octanoic acid, ethyl ester	n.d.	0.20 ± 0.06^a^	n.d.	n.d.	0.85 ± 0.08^b^	0.64 ± 0.05^a^
Hexanoic acid, ethyl ester	0.47 ± 0.06^a^	0.34 ± 0.04^a^	n.d.	n.d.	n.d.	n.d.
Benzoic acid, ethyl ester	0.53 ± 0.11^a^	n.d.	n.d.	n.d.	n.d.	n.d.
Acetic acid, 2‐phenylethyl ester	0.37 ± 0.10^a^	n.d.	n.d.	n.d.	n.d.	n.d.
Benzenepropanoic acid, methyl ester	0.32 ± 0.03^a^	n.d.	n.d.	n.d.	n.d.	n.d.
Benzenepropanoic acid, ethyl ester	0.78 ± 0.08^c^	0.24 ± 0.06^a^	0.35 ± 0.07^b^	1.76 ± 0.09^b^	1.65 ± 0.08^b^	1.37 ± 0.04^a^
Alcohols						
3‐Hexen‐1‐ol	0.44 ± 0.06^b^	0.36 ± 0.05^a^	0.31 ± 0.06^a^	n.d.	n.d.	n.d.
Benzyl alcohol	2.28 ± 0.53^c^	0.42 ± 0.06^a^	0.97 ± 0.16^b^	4.27 ± 0.36^b^	4.85 ± 0.12^b^	2.97 ± 0.36^a^
1‐Octanol	0.24 ± 0.04^a^	n.d.	n.d.	0.65 ± 0.02^a^	n.d.	0.61 ± 0.06^a^
Phenylethyl alcohol	7.02 ± 0.81^c^	2.64 ± 0.54^a^	6.56 ± 0.55^b^	25.93 ± 0.54^b^	27.44 ± 0.17^c^	22.78 ± 0.82^a^
Benzeneethanol, 4‐hydroxy‐	n.d.	n.d.	n.d.	4.07 ± 0.35^b^	n.d.	3.74 ± 0.59^a^
Aldehydes						
Benzaldehyde	n.d.	n.d.	5.78 ± 0.46^a^	n.d.	n.d.	0.64 ± 0.15^a^
Benzeneacetaldehyde	n.d.	1.07 ± 0.17^a^	1.04 ± 0.07^a^	n.d.	n.d.	3.31 ± 0.49^a^
Nonanal	0.83 ± 0.08^a^	n.d.	n.d.	3.07 ± 0.22a	n.d.	5.03 ± 0.34^b^
Vanillin	0.71 ± 0.15^b^	0.30 ± 0.07^a^	0.33 ± 0,05^a^	1.06 ± 0.05b	0.86 ± 0.09^a^	1.41 ± 0.03^b^
Phenol	0.46 ± 0.05^b^	0.30 ± 0.08^a^	0.34 ± 0.11^a^	n.d.	n.d.	n.d.
2‐Dodecenal	n.d.	n.d.	n.d.	n.d.	n.d.	0.35 ± 0.12^a^
Decanal	n.d.	n.d.	n.d.	0.42 ± 0.02^a^	0.52 ± 0.04^b^	1.12 ± 0.14^c^
2,4‐Decadienal, (*E*,*E*)‐	n.d.	n.d.	n.d.	0.43 ± 0.02^b^	0.40 ± 0.02^b^	0.33 ± 0.12^a^
2‐Undecenal	n.d.	n.d.	n.d.	n.d.	n.d.	1.06 ± 0.11^a^
Phenols						
Phenol, 2‐methoxy‐	7.67 ± 0.77^b^	3.09 ± 0.45^a^	3.65 ± 0.10^a^	4.88 ± 0.17	3.08 ± 0.08^b^	2.01 ± 0.29^a^
Phenol, 4‐ethyl‐	3.58 ± 0.87^c^	0.38 ± 0.04^a^	2.34 ± 0.09^b^	7.51 ± 0.1	3.33 ± 0.03^b^	2.99 ± 0.19^a^
Phenol, 2‐methoxy‐4‐methyl‐	12.17 ± 0.63^b^	7.27 ± 0.48^a^	17.03 ± 0.69^c^	n.d.	n.d.	n.d.
Guaiacol	n.d.	n.d.	n.d.	32.91 ± 0.57^c^	28.67 ± 0.49^b^	21.60 ± 0.60^a^
1,2‐Benzenediol	n.d.	n.d.	n.d.	n.d.	n.d.	1.16 ± 0.07^a^
Eugenol	n.d.	n.d.	n.d.	0.82 ± 0.04^b^	0.50 ± 0.02^a^	1.23 ± 0.26^c^
Lactones						
Butyrolactone	0.42 ± 0.05^a^	0.12 ± 0.03^a^	n.d.	n.d.	n.d.	0.29 ± 0.04^a^
Heterocyclic						
Pyridine, 3‐ethyl	1.12 ± 0.08^a^	n.d.	n.d.	n.d.	n.d.	n.d.
Benzothiazole	n.d.	n.d.	n.d.	n.d.	n.d.	0.51 ± 0.07^a^
Aromatic hydrocarbons						
Benzene, 1,3‐bis(1,1‐dimethylethyl)‐	0.17 ± 0.01^a^	0.42 ± 0.08^b^	0.17 ± 0.04^a^	n.d.	n.d.	n.d.
Naphthalene	n.d.	0.31 ± 0.10^a^	0.48 ± 0.04^b^	n.d.	n.d.	n.d.
Terpenes						
*β*‐Guaiene	n.d.	n.d.	0.19 ± 0.01^a^	n.d.	n.d.	n.d.
Copaene	1.38 ± 0.06^b^	1.20 ± 0.25^b^	1.02 ± 0.05^a^	0.59 ± 0.02^a^	0.54 ± 0.02^a^	0.61 ± 0.06^b^
*α*‐Farnesene	n.d.	0.69 ± 0.06^a^	3.13 ± 0.24^b^	n.d.	n.d.	n.d.
Other						
Methoxyphenyl oxime	1.44 ± 0.06^a^	3.36 ± 0.40^b^	7.99 ± 0.65^c^	6.74 ± 0.23^a^	20.20 ± 0.18^b^	21.97 ± 0.71^c^
**Total compounds**	**21**	**20**	**15**	**17**	**16**	**24**

Ne Ctrl, Nocellara Etnea spontaneously fermented; Ne LAB, Nocellara Etnea with *Lpb*. *plantarum* C11C8; Ne LAB+BSH, Nocellara Etnea with *Lpb*. *plantarum* C11C8 and *Lcb. rhamnosus* VB1; Ti Ctrl, Tonda Iblea spontaneously fermented; Ti LAB, Tonda Iblea with *Lpb. plantarum* C11C8; Ti LAB+BSH, Tonda Iblea with *Lpb. plantarum* C11C8 and *Lcb. rhamnosus* VB1. ^a−c^ Different letters within the same row indicate significant differences at *P* < 0.05; among samples of the same cultivar, values labeled with ‘a’ represent the lowest values, while subsequent letters indicate increasing values.

### Sensory profile and overall acceptability

The sensory profile of fermented olive samples based on the cultivar (Ti *versus* Ne) and on treatment (LAB *versus* LAB+BSH) is presented in Table 7.

**Table 7:** Sensory profile, expressed as mean ± standard deviation, of olive samples after 240 days

Sensory item	Cultivar		Std error	*P*	Treatment				Std error	*P*	RS‐square Adj
	Ti	Ne			Ti LAB	Ti LAB + BSH	Ne LAB	Ne LAB + BSH			
Green color°	2.94^b^	3.24^a^	0.12	0.027*	3.06	2.82	3.15	3.33	0.15	0.290	0.31
Odor intensity°	2.67^b^	3.18^a^	0.14	0.002*	2.58	2.76	3.36	3.00	0.18	0.198	0.36
Crispness°	3.53^b^	4.03^a^	0.13	0.001*	3.42	3.63	4.21	3.85	0.17	0.152	0.34
Taste intensity°	3.59^b^	3.97^a^	0.13	0.009*	3.45	3.72	3.93	4.00	0.16	0.388	0.37
Bitterness°	2.71^b^	3.41^a^	0.15	0.000*	2.67^b^	2.76^b^	3.72^a^	3.09^b^	0.20	0.046*	0.35
Overall acceptability°°	7.27^a^	6.09^b^	0.24	<0.001*	7.06^ab^	7.48^a^	5.70^c^	6.48^b^		0.05*	0.47

°Assessor scale: 1, low; to 5, high; °°assessor scale: 1, unsatisfactory; to 10, excellent; *P < 0.05. ^a–c^ Different superscripts within rows mean significant differences (*P <* 0.05); values labeled with ‘a’ represent the lowest values, while subsequent letters indicate increasing values.

Results revealed that the cultivar, mainly Ne, significantly influenced all parameters (*P* < 0.05). The inoculum of strains influenced (*P* < 0.05) bitterness and overall acceptability. Color intensity was rated as moderate across all samples (mean: 2.82–3.33). Samples inoculated with starter and probiotic strain (especially Ne) exhibited a slight increase in color intensity compared to the corresponding probiotic‐free controls. This effect is likely due to improved color stability during fermentation, which is probably influenced by the metabolic activities of the probiotic strain.

Odor intensity was slightly higher in samples without the probiotic strain, where the biotransformation of volatiles could produce a less intense but potentially more balanced aromatic profile. Texture (crispness) was the most highly rated parameter, with average scores exceeding 3.5 in all samples. Samples without the probiotic strain achieved slightly higher scores. This may reflect the impact of the probiotic strain on the cellular structure of olives, likely related to changes in the metabolism of structural sugars. Overall, samples with probiotic addition obtained higher scores for taste intensity compared to their probiotic‐free counterparts. This positive effect can be attributed to the ability of *Lcb*. *rhamnosus* VB1 to produce aromatic compounds that enrich the final flavor profile. Samples without the probiotic strain exhibited a more intense bitterness, whereas in samples with the probiotic strain, bitterness was moderate for both cultivars, suggesting a certain effect of the probiotic in a more balanced flavor. Samples with the probiotic received higher overall ratings, indicating greater overall acceptability, and improving the overall perception of the product. Ti samples obtained higher scores for all sensory attributes, confirming the strong potential for the Ti cultivar to be processed into table olives.

## DISCUSSION

Several studies have already indicated that specific starter cultures can enhance the debittering process and minimize the risk of harmful or spoilage microorganisms in table olive production.^40^ Furthermore, it has been widely documented that the microbial dynamics of fermentation are closely related to the processing technology applied, such as the reduction of salt content.^4^, ^17^, ^41^ Among the most effective strategies to lower the sodium content in table olives, the use of selected strains with *β*‐glucosidase activity has been proven promising for ensuring a safer and healthier product, in line with the recommendations of the WHO.^5^, ^17^, ^30^, ^42^

The present study investigated the effect of a selected strain of *β*‐glucosidase‐positive *Lpb. plantarum* (C11C8) on the microbial composition of Sicilian table olives, fermented at a reduced salt concentration (7%), for 150 days. It is well known that the *β*‐glucosidase enzyme plays a crucial role in the hydrolysis of oleuropein, facilitating the debittering process. Based on previous findings,^17^, ^20^, ^41^ the autochthonous *β*‐glucosidase‐positive strain was exploited as a starter to guarantee both the microbiological safety and the nutritional trait of two Sicilian olive cultivars. Overall, results revealed that the experimental table olives exhibited a more pronounced decrease in pH by the end of fermentation, reaching values ≤3.68 and ≤4.28 in Ti and Ne samples, respectively. Such faster brine acidification, compared to the control samples, enhances the microbiological safety of the final product, in agreement with previous studies.^43^, ^44^ Samples inoculated with the *Lpb. plantarum* starter showed a significantly shorter fermentation time and the inhibition of spoilage microorganisms.^45^ Microbiological data indicated a significant reduction in staphylococci populations starting from day 60 of fermentation, with values dropping below the detection limit by day 150. As reported in previous studies,^4^, ^17^ a significant decrease in Enterobacteriaceae was observed in inoculated brine samples as early as 30 days of fermentation. Notably, as already reported, our results highlighted a high prevalence of yeasts as part of the natural microbiota of table olives, coexisting with LAB throughout the whole fermentation process.^10^ Yeasts were particularly abundant in the Ne cultivar, and this may be linked to the intrinsic characteristics of the cultivar itself.^46^ Yeasts play a beneficial role in promoting bacterial growth, enhancing lactic acid production and inhibiting spoilage microorganisms, while contributing to the flavor and texture of the final product.^10^ In this study, yeast populations exhibited variable trends, with significantly high cell densities after 30 days of fermentation. In LAB‐inoculated samples, yeast populations were generally lower than in controls.

In recent years, growing consumer interest in functional foods has driven the food industry to explore innovative alternatives beyond traditional products, and table olives represent a promising option serving as natural carriers for probiotic microorganisms.^2^, ^29^ A critical aspect in developing a probiotic olive‐based product is the selection of strains able to adhere to the fruit surface and remain viable over an extended period. To be successfully employed, a microorganism must possess pro‐technological characteristics compatible with the production process while ensuring its viability, up to the end of shelf life. In this study, the probiotic *Lcb. rhamnosus* VB1, isolated from a human source, already characterized for BSH activity,^34^ probiotic and safety traits, according to EFSA,^47^ was successfully applied. Indeed, the qPCR analysis applied here provided a rapid, sensitive and highly specific quantification of strain density.^48^ In detail, the tracking tool revealed that the probiotic strain survived in table olives, resisting the stressful conditions and maintaining a concentration of about 6 log CFU mL^−1^, considered adequate, according to FAO/WHO guidelines for fermented foods.^49^, ^50^

Table olives, because of their natural content of phenolic compounds, are recognized as ‘functional foods’.^51^ Nevertheless, during storage, a decrease in total phenolic compounds and, in turn, a reduction in antioxidant activity have been described. Othman and co‐workers^52^ found that the concentration of phenolic compounds and antioxidant activity decreased even during the ripening stages of fruits. However, several studies have confirmed that the use of starter, particularly belonging to the *Lpb. plantarum* species, results in a significant increase in TPC. This phenomenon has been related to the activity of both *β*‐glucosidase and esterase enzymes on the main phenolic compounds present in olives, such as oleuropein, which is hydrolyzed into smaller molecules that, in turn, lead to an increase in TPC.^20^ Accordingly, results of the present study highlighted a marked increase in TPC during fermentation, with significant differences among different samples (*P* < 0.05). In particular, the addition of *Lpb. plantarum* strain C11C8 in Ne LAB and Ti LAB significantly affected the phenolic content, especially in Ti cultivar, where the initial value, right after 30 days, increased by 41.33 times and after 60 days by 43.53 times. In Ne cultivar, the initial value increased by 22.3 times after 150 days. These data suggest that fermentation can differently affect the kinetics of release or transformation of phenolic compounds based on the cultivar.^46^, ^53^ Interesting results were obtained in samples added with the probiotic strain *Lcb. rhamnosus* VB1 (Ne LAB+BSH and Ti LAB+BSH), where a greater increase in phenolic content up to 240 days was detected, compared to samples inoculated with the starter alone or spontaneously fermented.

According to previous studies, a positive correlation between TPC and RSA has been found in different foodstuffs, such as vegetables and fruits.^54^, ^55^ In the present study, the results of RSA, evaluated by the DPPH radical scavenging assay, confirmed the correlation with TPC. At the end of fermentation, Ti LAB+BSH showed slightly higher RSA than Ti Ctrl, suggesting a stable maintenance of antioxidant capacity, preventing its decline, according to previous findings.^56^, ^57^ Further studies are required to explore the role of microbiota in modulating the functional properties of table olives.^40^, ^58^ Results on the sensory profile demonstrated that the addition of *Lcb. rhamnosus* VB1 significantly modified the sensory attributes of final products. A noticeable improvement in the overall flavor profile was observed, with a reduction in bitterness and an increase in consumer acceptance. The intensity of aromas and color was influenced by the probiotic, suggesting the possibility of modulating these attributes according to specific market preferences through the optimization of fermentation conditions. Texture, a crucial parameter for the perceived quality, remains high in all tested samples, confirming the effectiveness of the fermentation process. However, the slight differences observed in samples inoculated with the probiotic strain suggest further investigations into strain selection. Results of the present study confirmed that the addition of *Lpb. plantarum* and *Lcb. rhamnosus* VB1 profoundly reshaped the VOC profile, enhancing the production of desirable aromatic compounds and significantly reducing off‐flavor molecules. In contrast, non‐inoculated samples showed a higher content of alcohol and phenol compounds associated with bitter and pungent notes, such as 4‐ethylphenol, as previously reported.^20^

A correlation between sensory data and VOC profiles was found. LAB samples exhibited a more intense odor compared to LAB+BSH, suggesting that the probiotic can modulate the perception of odor intensity. Regarding taste intensity, LAB+BSH samples received higher scores for overall taste. As reported,^59^ the fermentation process using selected lactobacilli enhances a product's overall perception, particularly in acidity and bitterness. Samples with the probiotic showed a better balance of sweetness, acidity and bitterness. In these samples, the addition of the probiotic led to a more harmonious interplay of these flavors. Accordingly, Ne LAB+BSH samples showed unique or increased levels of compounds such as *α*‐farnesene and 2‐methoxy‐4‐methylphenol.

For both cultivars, LAB samples showed higher levels of propanoic acid, which contributes to a pungent acidic taste. It is interesting to observe that in both Ne Ctrl and Ti Ctrl samples, a higher presence of 4‐ethylphenol, a phenolic compound considered an off‐flavor, generally produced by microorganisms through the decarboxylation of *p*‐coumaric acid into 4‐vinylphenol and its subsequent reduction,^60^ was observed. Based on this finding, the probiotic strain seems to inhibit its production. These findings suggest that the modulation of VOC profiles through targeted microbial inoculation can be strategically used to meet different consumer preferences. Indeed, the increase in esters, such as ethyl benzoate, in probiotic‐enriched samples may enhance fruity and sweet aromatic notes, contributing to a more balanced taste and masking residual bitterness – an attribute often associated with higher consumer liking. Conversely, the reduction of off‐flavor compounds like 4‐ethylphenol, commonly perceived as undesirable, can improve the overall sensory acceptance of the product. The ability to reduce pungent acidic notes (e.g. from propanoic acid) while enhancing pleasant aroma compounds offers the possibility of tailoring flavor profiles to specific market segments, from traditional consumers accustomed to robust flavors to new consumers seeking milder, more approachable products. These observations strengthen the connection between analytical VOC profiles, sensory perception and their potential impact on consumer acceptance and market positioning.

## CONCLUSION

The present study proposes a biotechnological approach to enhance natural green table olives' safety and sensory traits, optimizing the fermentation process and enriching the product with a selected probiotic strain. Results exhibited the ability of the probiotic strain to survive while maintaining the chemical, microbiological and sensory characteristics of table olives. From an industrial perspective, the use of a validated microbial starter proved to be effective in enhancing product acidification and reducing bitterness, while ensuring safety through microbial competition. This competitive activity contributed to lowering the presence of spoilage and potentially pathogenic microbial populations, even within a low‐sodium brine fermentation. The feasibility of introducing targeted probiotic strains into large‐scale production without compromising sensory traits represents a valuable innovation for producers, meeting the growing demand for healthier, sustainable and microbiologically safe plant‐based foods, while remaining consistent with traditional processing methods. The results will serve as the foundation for designing a lower‐sodium functional food capable of delivering beneficial probiotic microorganisms, opening new perspectives in the functional food sector.

## AUTHOR CONTRIBUTIONS

**Irene M. Zingale:** investigation, data curation, writing – original draft preparation; **Amanda Vaccalluzzo:** methodology, visualization; **Giacomo Antonio Calandra Checco:** investigation, data curation; **Vita Maria Marino:** methodology, writing – original draft preparation; **Margherita Caccamo:** writing – review and editing, visualization, data curation; **Cinzia L. Randazzo:** methodology, data curation, supervision; **Russo Nunziatina:** investigation, data curation, visualization; **Dilara Nur Dikmetas**: investigation, data curation; **Tuba Esatbeyoglu**: visualization, supervision, funding acquisition; **Esra Capanoglu:** methodology, project administration; **Cinzia Caggia:** data curation, resources, visualization, funding acquisition.

## FUNDING INFORMATION

This research is part of a project (Oli4food) that has received funding from the PRIMA Programme PRIMA Section2 – Multi‐topic 2022: CUP no. E93C23000230007, supported by the European Union's Horizon 2020 Research and Innovation Programme, project ID no. 1854.

## CONFLICTS OF INTEREST

The authors declare no conflict of interest.

## Data Availability

The data that support the findings of this study are available from the corresponding author upon reasonable request.
